# Secondary Psychosis Following Neoadjuvant AC-T Chemotherapy for Triple-Negative Breast Cancer: Case Report and Literature Review of Psychosis Postchemotherapy

**DOI:** 10.1155/2022/4939219

**Published:** 2022-10-28

**Authors:** Sultan Alshehri, Hatem Assiri, Moayyad Alsalem, Majed A. Alharbi

**Affiliations:** ^1^College of Medicine, King Saud Bin Abdulaziz University for Health Sciences, Riyadh, Saudi Arabia; ^2^King Abdullah International Medical Research Center, Riyadh, Saudi Arabia; ^3^Department of Adult Mental Health, King Abdulaziz Medical City, Ministry of the National Guard-Health Affairs, Riyadh, Saudi Arabia; ^4^Psychiatry Section, Department of Medicine, King Abdulaziz Medical City, Ministry of the National Guard-Health Affairs, Jeddah, Saudi Arabia; ^5^King Abdullah International Medical Research Center, Jeddah, Saudi Arabia; ^6^College of Medicine, King Saud Bin Abdulaziz University for Health Sciences, Jeddah, Saudi Arabia

## Abstract

Triple-negative breast cancer is a unique subtype among breast cancers. Management includes a neoadjuvant chemotherapy regimen. Psychiatric complications of the regimen have not been reported before. We present a case of acute psychosis after the second cycle of chemotherapy in a 42-year-old woman with triple-negative breast cancer. The patient presented with sudden irritability, agitation, disorganization in speech and behavior, and paranoia involving her coworkers conspiring against her and causing her trouble with the law for 4 days. She was in her usual state of health until after her second cycle of chemotherapy. This was the first presentation of psychotic symptoms in her life. She was conscious and oriented. There were no neurologic deficits. She denied any change in her mood and any features of hallucinations. She was uncooperative, restless, had flight of ideas, and persecutory delusions. The remainder of the examination was normal. An autoimmune process, nervous system infection, or psychosis secondary to the chemotherapy were suspected. Serum electrolytes and other biochemical parameters were normal. Imaging of the brain showed no signs of acute brain insults or intracranial metastasis. Cerebrospinal fluid analysis and culture showed no abnormality or growth. The work-up revealed that neurologic, infectious, or autoimmune causes of her psychotic symptoms were less likely. Thus, a diagnosis of psychosis secondary to chemotherapy was considered. Treatment was with paliperidone, risperidone, clonazepam, and sertraline. Over the course of treatment, she showed substantial improvement and completed all of the chemotherapy sessions without adverse effects. In summary, we report a case of a patient whose initial chemotherapy course was complicated by psychosis. Since the neurotoxic and psychiatric effects of chemotherapeutics are not yet sufficiently elucidated, our case emphasizes that early signs of behavioral changes in patients receiving chemotherapy should trigger comprehensive psychiatric evaluation and monitoring of the patient's mental state.

## 1. Introduction

Breast cancer is the most common cancer diagnosed among women [1]. Triple-negative breast cancer (TNBC) is a subtype of breast cancer that, based on immunohistochemistry, is estrogen receptor negative, progesterone receptor negative, and human epidermal growth factor receptor 2 negative. TNBC is characterized by its unique molecular profile, aggressive nature, distinct metastatic patterns, and lack of targeted therapies. And it accounts for 10-20% of invasive breast cancers [2].

Patients who have suffered from TNBC are offered a neoadjuvant chemotherapy regimen that comprises of dose-dense doxorubicin and cyclophosphamide plus docetaxel (AC-T) [3–5]. Complications and side effects from neoadjuvant AC-T chemotherapy, such as peripheral neuropathy, neutropenia, and amenorrhea, have been commonly described in the literature [6]. Yet, psychiatric complications have not been reported. We present a case of acute psychosis appearing after the second cycle of dose-dense neoadjuvant AC-T chemotherapy in a 42-year-old woman who was diagnosed with TNBC.

## 2. Literature Review of Secondary Psychotic Symptoms Postchemotherapy

There have been previous reports in the literature regarding cancer patients developing psychotic symptoms while or after undergoing chemotherapy. Garg et al. reported a case of secondary mania following the 3^rd^ cycle of chemotherapy of a 46-year-old man with stage 3C colon carcinoma. The patient underwent surgical resection with diversion ileostomy then chemotherapy with a combination of capecitabine and oxaliplatin. Shortly after the 3^rd^ chemotherapy cycle, the patient started showing increasing signs of irritability, anger outbursts, overtalkativeness, decreased need for sleep, increased goal-directed activity, increased appetite, heightened self-esteem, and overreligiosity. Serum electrolytes and other biochemical parameters were found to be within normal range. MRI brain showed features suggestive of mild cerebellar and cerebral atrophy and calcification in bilateral basal ganglia region, yet the authors concluded that it did not explain the acute presentation of the patient [7]. Another report by Campbell and Panicker involved a 16-year-old male with left testicular nonseminomatous germ cell tumor that developed psychotic symptoms after the 2^nd^ cycle of chemotherapy. The patient underwent radical left orchiectomy then was planned for two cycles of chemotherapy involving bleomycin, etoposide, and cisplatin (BEP). After the 1^st^ chemotherapy cycle, mental status changes were noted by his parents. Over the next few days, his parents noticed that his behavior and thoughts became increasingly bizarre and grandiose. He also began having racing thoughts, restlessness, and insomnia. On evaluation by psychiatry, he was noted to be delusional, grandiose, and displayed features of agitation. Similar to Garg et al., physical examination, biochemical markers and parameters, and brain imaging were unremarkable [8].

Another report by Puangthong and Pongpirul involved a 25-year-old Thai woman with ovarian germ cell tumor that developed psychotic symptoms after the last cycle of a 3 cycle chemotherapy regimen of BEP. A week after the last cycle and being discharged from the hospital, her behavior had been increasingly disorganized and bizarre. She could not remember her mother and went to her neighbor's house thinking it was hers. She was irritable and hyperactive. She displayed delusions that the king had transferred 50 million baht into her bank account. Moreover, she displayed sudden impulsive behavior in the form of spending a lot of money buying things. She also displayed grandiosity as well as some paranoia. Similar to the abovementioned cases, physical examination, biochemical markers and parameters, and brain imaging were unremarkable [9]. Pavlova and Weinrebe reported a case of a 73-year-old female patient with multiple myeloma developing psychotic symptoms during chemotherapy cycles. The patient underwent chemotherapy with Velcade-Revlimid-Dexamethasone-based treatment. After starting chemotherapy, the patient's family noticed some changes in her mood, sleep disturbances, and significantly worsened cognitive impairment. One week after the first chemotherapy cycle, the symptoms became more prominent, and the patient was admitted again for cognitive assessment. On physical examination, the patient was alert, markedly elated, agitated, hyperactive and very talkative, and totally disoriented to place and time but oriented to person and in acute pain. The patient had episodes of elevated and excited mood, increased energy, restlessness, decreased sleep, and pressured speech. Moreover, persecutory delusion, grandiose delusion, and conversations with voices were suspected. Findings of brain imaging of the brain were not remarkable in context of this patient's new onset of psychotic symptoms. Neurological examinations and laboratory data showed no abnormality. A neuropsychological examination revealed moderately severe disorders in attention, comprehension, severe impairment in episodic memory, and executive function [10].

## 3. Case Presentation

Two months prior to the presentation, the patient had visited the emergency department complaining of a painful left breast swelling for 5 days. A mammogram was done, which then showed a highly suspicious BI-RADS 5 mass. Subsequently, a core needle biopsy was performed and revealed a grade 3, Ki-67 50%, invasive ductal carcinoma of the “triple negative” phenotype. TNM staging was T2N0M0. She was then scheduled to receive 4 biweekly cycles of neoadjuvant chemotherapy of dose-dense AC followed by 4 cycles of docetaxel.

One week after she had her second cycle of AC-T, the patient was brought to the emergency department by her family members with a chief complaint of acute psychotic symptoms and insomnia for 4 days. Vital signs show a temperature of 36.9°C, the heart rate 97 beats per minute, the blood pressure 126/91 mm Hg, the respiratory rate 21 breaths per minute, and the oxygen saturation 97% while the patient was on room air. She was conscious and oriented to time, place, and location. The score on the Glasgow Coma Scale was 15. The patient was in her usual state of health; until a few days after the second cycle of AC, her family had noticed that the patient suddenly started showing flare-ups of her anxiety symptoms, irritability, and started to be agitated, showing disorganization in terms of speech and behavior and paranoia involving governmental institutions and her coworkers strongly believing that they are conspiring against her and causing her trouble with the law. Her disorganization was noticeable by the family and even later was noticeable when evaluated and admitted in the hospital. There was no history of headaches, blurry vision, seizures, nor weakness, and numbness in the limbs prior to the presentation. She denied any change in her mood and denied any features of hallucinations; however, it has been not ruled out. She also denied any suicidal or homicidal plans and ideations. She was uncooperative, restless, had flight of ideas, and persecutory delusions. The remainder of the examination was normal.

This was the first presentation of psychotic symptoms in the patient's life. Aside from her past psychiatric history of generalized anxiety disorder (GAD) and recent diagnosis of TNBC, the patient had not been ill recently and had no significant medical nor surgical history. The patient had no recent history of substance use, such as cannabis, nor has she ever been treated for substance use disorders. The patient had no known adverse reactions to medications and only took escitalopram for her GAD. She worked in a public school as a teacher and is a divorced mother of 3 that lives alone in her apartment. She was fully independent in her activities of daily living and was able to carry out her routine unburdened. Aside from her brother's previous history of depression and substance-induced psychosis, there was no known family history of psychiatric or other neurologic diseases. The patient was then admitted into the hospital under oncology. Psychiatry, neurology, and infectious diseases were then consulted and involved in her case under the impression that her acute presentation of psychotic symptoms was due an autoimmune process, central nervous system (CNS) infection, such as meningoencephalitis, or organic psychosis secondary to corticosteroid use or secondary to the chemotherapy regimen.

At the time of assessment by psychiatry, she was put under constant observation by the primary team and nurses due to the cyclical nature of her agitation and great deal of disorganization. She tried to leave the hospital on several occasions to report her housemaid and coworker to the police. Moreover, on psychiatric assessment, the patient was found to have lack of insight regarding her psychotic symptoms and was not able to govern herself safely. She has also been found to be suspicious, agitated, and not taking proper care of herself. Furthermore, she expressed paranoid ideation against hospital staff. On laboratory investigations, aside from mild leukocytosis, the patient had normal serum electrolyte levels with normal blood counts, liver function, kidney function, blood sugar levels, inflammatory markers, and urinalysis. Blood culture revealed *Neisseria flavescens* and *Streptococcus viridans* group bacteremia with no clear source of infection. Echocardiography was done on the suspicion of infective endocarditis, but the results were grossly normal and showed no signs of valvular pathology, infection, nor wall motion abnormalities. Repeat blood culture was negative for growth. MRI and CT of the brain were done, but they were grossly normal and showed no signs of acute brain insults or intracranial metastasis. Cerebrospinal fluid analysis was done, and the results showed no abnormality, and culture was negative for growth. Making a neurological, autoimmune, infectious, metabolic, paraneoplastic, or metastatic cause of her acute psychotic symptoms is less likely. Thus, a diagnosis of psychosis secondary to chemotherapy was considered.

Overall, she showed good judgement, decision-making capacity, and insight regarding her breast cancer diagnosis and treatment, but she had poor insight regarding her diagnosis of psychosis and refused to take any oral antipsychotic medications. The patient was treated with intramuscular olanzapine 10 mg twice per day. For restlessness and agitation, she was given, as needed, intramuscular lorazepam 2 mg, subcutaneous haloperidol 5 mg, and an additional dose of intramuscular olanzapine 5 mg. During the patient's stay, she completed her third cycle of AC chemotherapy without any adverse effects. Furthermore, the patient's clinical and mental state had dramatically improved over time. It was noticed that the patient prior to discharge was more cooperative, no longer felt restless, was less disorganized, and no longer expressed abnormal behavior. The patient still showed persecutory ideations but was not acting on delusions for the last week before discharge. She was then discharged with clonazepam 2 mg/day and a follow-up appointment with outpatient psychiatry. During outpatient visits, the patient still refused oral antipsychotic therapy and was prescribed sertraline for her symptoms of anxiety, as she had a previous history of GAD that was treated with escitalopram. During this outpatient course, sertraline was started with a dose of 25 mg once per day then gradually increasing to 100 mg once per day. No side effects were reported.

The patient completed all 4 cycles of AC chemotherapy and was then to be followed by 4 cycles of docetaxel. A week after the second cycle of docetaxel, the patient presented again to the emergency department with history of poor oral intake, vomiting, and documented fever of 38°C for a week. Additionally, similar to the last visit, presentation of psychotic symptoms including insomnia, agitation, and delusions of persecution were apparent. Physical, neurological, and mental status examinations were similar to the previous presentation. The patient was then admitted for work-up of the fever. Labs, culture, and imaging were unremarkable. The patient was treated with tazocin, and the fever subsided. On psychiatric evaluation, she had similar presentation of irritability, paranoid delusions, and disorganized behavior. The patient was treated with risperidone 3 mg and clonazepam 2 mg. For restlessness and agitation, she was given, as needed, intramuscular lorazepam 2 mg and subcutaneous haloperidol 2.5 mg. On discharge, the patient was given a depo intramuscular injection of 150 mg of paliperidone.

On subsequent follow-up visits during the outpatient treatment course, she was treated with paliperidone, risperidone, clonazepam, and sertraline. Paliperidone was started as a monthly 100 mg injection then increased to 150 mg monthly injection. Over time, she responded to paliperidone, and subsequently, risperidone 3 mg and clonazepam 1 mg were tapered until finally discontinued. During this outpatient course, the patient was restarted on sertraline again with a dose of 25 mg once per day then gradually increasing to 100 mg once per day, similar to the course before the 2^nd^ presentation. No side effects were noticed during course of treatment, though benztropine mesylate 1 mg was prescribed on an “as needed” basis in case of tremors or symptoms indicating extrapyramidal side effects. Over the course of treatment, she showed substantial improvement. She was less irritable overtime and had no active psychotic symptoms. The patient successfully completed all of the AC-T chemotherapy sessions. She then underwent nipple-areola complex lumpectomy with sentinel lymph node biopsy and inferior margin excision, both of which were negative for malignancy.

## 4. Discussion

To the best of our knowledge, this is the first report of psychosis occurring secondary to the AC-T chemotherapy regimen and the first report of psychosis among women with breast cancer undergoing chemotherapy in general. In this report, psychotic symptoms were noticed that was not related to an established psychiatric disorder or structural brain damage but occurred in temporal proximity to AC-T chemotherapeutic treatment of breast cancer. In these such cases, there is difficulty in differentiating between incidence of secondary psychosis and development of primary psychosis. However, in this report, the first episode of psychotic symptoms with acute onset occurring at 42 years of age, in the absence of family history of psychiatric disorders and with absence of any other neurological signs and symptoms or abnormal laboratory test results that suggest alternative causes, strongly suggests secondary psychosis to be the most probable case rather than a primary psychotic disorder.

Our patient underwent the AC-T chemotherapy regimen consisting of 4 cycles of dose-dense doxorubicin and cyclophosphamide followed by 4 cycles of docetaxel. She presented for the first time after the 2^nd^ cycle of the AC portion of the regimen and presented again for the 2^nd^ time after the 2^nd^ cycle of the docetaxel portion of the regimen. For both presentations, the patient was not actively taking any antipsychotic treatment. First, this may demonstrate that the chemotherapeutic agents in both portions of the regimen may have been responsible for the adverse psychiatric effects. Second, this implicates that the patient was especially susceptible to developing the adverse psychiatric effects of the regimen during those periods of absent antipsychotic treatment.

One point to note, when comparing our case to the 4 mentioned cases in the literature, all of the patient's mentioned, and their family members, had no past psychiatric history or decompensation, no mention of substance use, and were functional in their activities of daily living which may further suggest the temporal association between receiving chemotherapy and developing new-onset psychotic symptoms. One exception is the patient mentioned in the report by Campbell and Panicker. Significant findings include a history of traumatic brain injury and depression with suicidal ideation. Other significant findings were that his home life was disrupted by domestic violence between his divorced parents. The mentioned findings may suggest that psychosocial and family stressors may be considered factors contributing to higher psychiatric risks when undergoing chemotherapy. Another point to note that neurological and physical examination findings, biochemical markers and parameters, and brain imaging were unremarkable in our case and all of the patients reported, suggesting neurological, autoimmune, infectious, metabolic, paraneoplastic, and metastatic causes of their presentation to be less likely. A common factor between all of the patients is the development of new-onset psychotic symptoms during or days after treatment by chemotherapeutic agents, suggesting a temporal association between chemotherapy and development of psychosis. Moreover, the new-onset psychotic symptoms presented for the first time after the second or third cycle among most of the reported patients, which may suggest that the adverse effects of these chemotherapeutic agents may be cumulative or delayed. Another factor to consider is the presence of acute stressors in the form of patients being newly diagnosed with cancer and starting treatment with chemotherapy.

There is inherent difficulty with determining the cause of new-onset neurological or psychiatric presentations among cancer patients undergoing chemotherapy. Many types of cancers are known to cause nonspecific neurological or psychiatric effects in some cases, even without chemotherapy, for example, the different paraneoplastic syndromes that can affect the CNS of patients without direct tumor invasion. The neurological or psychiatric presentations of these syndromes demonstrate different signs and symptoms depending on the region of the brain affected [11]. Thus, there may be overlap with the symptoms of the paraneoplastic syndromes and the neurological or psychiatric symptoms of adverse chemotherapy reactions, making the process of differentiating between them difficult. Moreover, many forms of cancers are treated with fixed drug regimens, which make it difficult to isolate which specific chemotherapeutic agent is responsible. Among all the cases reported, in terms of specific types of cancer and its location being a factor, the two patients reported by Puangthong and Pongpirul and Campbell and Panicker were both treated for gonadal germ cell tumors. The rest of the reported patients was treated for different forms of cancer involving different systems and organs, which may indicate that specific types of cancer and its location does not seem to be especially implicated with development of new-onset psychotic symptoms. Similarly, in terms of specific chemotherapeutic agents, only the two patients reported by Puangthong and Pongpirul and Campbell and Panicker were treated with the same BEP chemotherapeutic regimen consisting of bleomycin, etoposide, and cisplatin. All other reported patients underwent different chemotherapy regimens. However, some of the regimens included similar chemotherapeutic agents. The regimen for the patient reported by Garg et al. included oxaliplatin, an analog of cisplatin, and both platinum-based chemotherapeutic agents. In a study examining the neurotoxic effects of chemotherapeutic agents associated with varying levels of blood-brain barrier permeability, it has been demonstrated that platinum-based agents and doxorubicin were highly neurotoxic. However, cyclophosphamide was not associated with substantial neurotoxicity. It was also demonstrated that the lack of neurotoxicity associated with the usual administration of these agents was most likely related to their limited entry into the CNS through an intact blood-brain barrier [12]. It has been suggested that the blood-brain barrier among cancer patients is compromised, which may provide another mechanism of entry into the CNS for neurotoxic chemotherapeutic agents [13]. Platinum-based agents and doxorubicin demonstrate poor penetration through the blood-brain barrier [14, 15]. Yet, levels of doxorubicin were found to be elevated in the CNS when coadministered with certain medications, e.g., proton pump inhibitors, calcium channel blockers, amiodarone, and certain selective serotonin reuptake inhibitors such as sertraline and paroxetine. And this effect is dose dependent [16, 17]. This may suggest a possible alternative mechanism if the blood-brain barrier has been passed to a sufficient extent, potentially causing CNS neurotoxicity.

Our patient and all of the other 4 reported patients started treatment with olanzapine, except for the patient reported by Campbell and Panicker who was instead started on risperidone and became stable over time with remittance of psychotic symptoms. The patient reported by Garg et al. continued on olanzapine and symptoms subsided after 4 weeks. For the patient reported by Puangthong and Pongpirul, olanzapine failed to control psychotic symptoms during the first week and was therefore switched to risperidone. Her symptoms were dramatically controlled within the second day of risperidone. Similarly, for the patient reported by Pavlova and Weinrebe, olanzapine failed to control psychotic symptoms and was then started haloperidol in combination with diazepam and quetiapine. By the third week, psychotic symptoms had remitted. In comparison, our patient was started initially on olanzapine during the first admission after receiving the second cycle of AC chemotherapy and symptoms subsided overtime. When the patient was admitted again after receiving the second cycle of docetaxel, the patient was started on olanzapine but was switched to paliperidone and risperidone on discharge. All patients mentioned on follow-up had remittance of psychotic symptoms and are stable. This may indicate that discontinuation of chemotherapy and start of antipsychotic treatment may have reversed the reported patients' psychopathological state. Hence, our case and the above cases highlight the importance of pharmacovigilance in chemotherapy with regard to development of psychiatric symptoms as these may affect compliance to treatment and, thus, long-term prognosis.

## 5. Conclusion

In summary, we report a case of a middle-aged female patient whose initial chemotherapy treatment with AC-T for TNBC was complicated by development of new-onset psychosis. The patient's presentation at the age of 42 is atypical for primary psychotic illness, and she had been functional beforehand with no acute psychiatric decompensation in her life. Since neurotoxic and psychiatric effects of chemotherapeutics are not yet sufficiently elucidated, our case emphasizes that early signs of behavioral changes in patients receiving chemotherapy should trigger comprehensive psychiatric evaluation and ongoing monitoring of the patients' mental state. Moreover, other factors such as acute stressors in the context of being newly diagnosed with cancer and subsequently starting chemotherapy should factor-in together with the suspected temporal association between chemotherapy and psychosis. For our patient, during our first encounter with her, antipsychotic treatment was delayed as our focus at the time was to verify and establish a diagnosis by excluding any neurological or active medical causes that could have contributed towards her first presentation of psychotic symptoms. Yet, we realize it would have been beneficial if we had started antipsychotic treatment immediately once the patient presented for the first time. We maintained her on antipsychotic treatment for most of the duration of her chemotherapy course, and she showed good response and was compliant to the chemotherapeutic regimen. As the continuity of chemotherapeutic treatment is essential and interrupting the course may lead to unfortunate oncological decompensation, we suggest starting and continuing antipsychotic treatment for the entire duration of a psychotic patient's chemotherapeutic treatment course.
